# Bromelain and Nisin: The Natural Antimicrobials with High Potential in Biomedicine

**DOI:** 10.3390/pharmaceutics14010076

**Published:** 2021-12-29

**Authors:** Urška Jančič, Selestina Gorgieva

**Affiliations:** 1 Institute of Engineering Materials and Design, Faculty of Mechanical Engineering, University of Maribor, Smetanova ulica 17, 2000 Maribor, Slovenia; urska.jancic@um.si; 2 Institute of Automation, Faculty of Electrical Engineering and Computer Science, University of Maribor, Koroška cesta 46, 2000 Maribor, Slovenia

**Keywords:** bromelain, nisin, bioactivity, antimicrobial agent, biomedicine, carrier

## Abstract

Infectious diseases along with various cancer types are among the most significant public health problems and the leading cause of death worldwide. The situation has become even more complex with the rapid development of multidrug-resistant microorganisms. New drugs are urgently needed to curb the increasing spread of diseases in humans and livestock. Promising candidates are natural antimicrobial peptides produced by bacteria, and therapeutic enzymes, extracted from medicinal plants. This review highlights the structure and properties of plant origin bromelain and antimicrobial peptide nisin, along with their mechanism of action, the immobilization strategies, and recent applications in the field of biomedicine. Future perspectives towards the commercialization of new biomedical products, including these important bioactive compounds, have been highlighted.

## 1. Introduction

One of the tremendous burdens on human health worldwide is infectious diseases [1], where antibiotics act as first-line therapy in treating infections caused by bacteria. Still, their widespread use, over-utilization and improper consumption in humans and animals cause an increase in the number of resistant bacterial strains. Furthermore, one pathogen organism is gaining resistance to more than one antibiotic, leading to the development of multidrug resistance strains for various species, such as *Staphylococcus aureus* (*S. aureus*), *Pseudomonas aeruginosa* (*P. aeruginosa*), *Salmonella* spp., *Enterococcus faecium* (*E. faecium*), *Campylobacter*, *Neisseria gonorrhoeae* (*N. gonorrhoeae*), *Streptococcus pneumonia* (*S. pneumonia*) [2], etc. Consequently, the cost of hospitalization and healthcare, together with morbidity and death are increasing [3]. According to World Health Organization and Organisation for Economic Co-operation and Development at least 700,000 patients die every year from infections caused by resistant microorganisms [2] and approximately 2.4 million people in Europe, North America, and Australia are expected to die due to diseases caused by drug-resistant pathogens over the next 30 years, which means $3.5 billion in economic cost per year [4]. Furthermore, multidrug resistance of cancer cells against conventional chemotherapeutic agents [5] is another problem that needs to be solved. Therefore, it is necessary to search for innovative alternative therapies and new drug candidates [6]. Various studies exhibit promising results when natural antimicrobial peptides and proteins are used as therapeutics [7], especially since their conjunction with conventional chemotherapeutic agents promotes effectiveness, decreases antibiotics use and possibly reduces instances of chemotherapy resistance [8].

This review gives a comprehensive overview on two compounds obtained from two different natural sources, i.e., nisin as a bacterial origin representative and bromelain as a plant origin representative. With nearly 50 years of safe usage in the food industry, and very little evidence of cross-resistance compared with that of conventional antibiotics [7,8], non-toxicity and low immunogenicity [9], researchers have begun to explore the nisin, an antimicrobial peptide with a broad-spectrum of antibacterial activity [6] as a potential alternative agent for infectious diseases [7]. On the other hand, the demand for medicinal plants with therapeutic agents has been rising [10] as natural plant products are increasingly recognized as non-toxic, side-effect free, readily available and affordable [1]. Among them, pineapple has been identified to possess valuable qualities for medical purposes, especially its proteolytic enzyme bromelain due to its antimicrobial, anti-inflammatory, anti-thrombotic, fibrinolytic and anti-cancer functions [11]. The present review comprehensively discusses the structure, isolation and suggested bioactivity mechanisms, as well as immobilization strategies and application of nisin and bromelain in the last 10 years. Published reports were collected using the Web of Science and Scopus databases, with search terms “bromelain”, “nisin”, “bioactive”, “antimicrobial”, “anticancer”, “anti-inflammatory”, “toxicity”, “immobilization”, “adsorption”, “encapsulation”, “entrapment” and “carrier”. Our aim is to emphasize the importance and relevance of these bioactive compounds, where the researchers and relevant stakeholders may gain the latest fundamental knowledge to explore the new possibility of bromelain- or nisin-based products in biomedicine and pharmacy. Moreover, giving comprehensive information for two different origin bioactive compounds can allow direct comparison of their ultimate properties and action, giving the ease of selecting the suitable candidate for a particular biomedical application. Relating to this, we also point to very limited clinical trials (and even fewer approved products) involving bromelain and nisin, as contradictory to the potential they hold in this segment. As a hypothetically written, future perspective, the possibility to combine both bioactive components in an attempt to merge and even boost their multiple bioactivities, utilising diverse immobilization routes, have been brought forward.

## 2. Bromelain

### 2.1. Structural and Biological Properties

Bromelain is a protein purified from a crude aqueous extract of pineapples (*Bromeliaceae* family) [1]. Pineapple is a common name of *Ananas comosus*, also known as *Ananassa sativa*, *A. sativus*, *Bromelia ananas* or *B. comosa*, grown in several (sub)tropical countries such as Costa Rica, Philippines, Brazil, Thailand, China, Indonesia, India, Malaysia, Hawaii and Kenya [1,12]. In the pineapple plant, bromelain acts as a defensive protein; it protects the pineapple throughout the development, maturation and ripening process [13,14].

Bromelain was identified for the first time in 1891 by Vicente Marcano, a Venezuelan chemist, while its isolation and analysis started in 1894. However, its commercial production began in 1957 with Heinecke’s discovery that the pineapple fruit contains less bromelain than the pineapple stem [15], making a waste by-product stem bromelain more commercialized [13].

Bromelain belongs to the class of proteases also known as proteinases or peptidases, a group of enzymes that catalyzes proteolytic reactions where the breakdown of proteins into smaller polypeptides or single amino acids occurs [13,16,17]. More specifically, it is classified as cysteine proteinase (EC 3.4.22, CP, also known as thiol proteinase) due to the cysteine thiol in its active site [1,13]. Crude bromelain (crude extract of the pineapple) contains various cysteine endopeptidases and other components, including phosphatases, glucosidase, peroxidases, cellulases, glycoproteins, carbohydrates, ribonucleases, protease inhibitors and organically bound calcium [1,12,15]. Among them, the specific activity of proteases is the highest, e.g., the specific activity of protease, peroxidase, acid phosphatase, alkaline phosphatase and amylase studied in the crude bromelain extracted from pineapple crown leaf was 45 U/mg, 2.19 U/mg, 1.12 U/mg, 0.98 U/mg, 0.65 U/mg, respectively [18]. At least four evolutionarily and structurally related cysteine endopeptidases can be synthesized from crude bromelain: stem bromelain (EC 3.4.22.32), fruit bromelain (EC 3.4.22.33), ananain (EC 3.4.22.31) and comosain (Table 1) [1,13,15]. Stem bromelain is the major protease present in the stem of the pineapple plant, and fruit bromelain is the major protease in the pineapple fruit [1,19]. Ananain and comosain were detected only in minor quantities in stem pineapple [1]. All the endopeptidases of the pineapple plant have generally been referred to as “the bromelains” and the name “bromelain” was originally used to describe any protease of the *Bromeliaceae* family [15].

All four cysteine endopeptidases possess distinguished physicochemical properties, as summarized in Table 1. Fruit bromelain is an acidic protein, unlike stem bromelain, which is alkaline (isoelectric point 4.6 and ≥9.5, respectively). Generally, the molecular weight of stem and fruit bromelain is from 23.8 to 37.0 kDa and 23.0 to 32.5 kDa, respectively. This heterogeneity in molecular weight may be due to heterogeneity of the amino acid sequence and the glycosylation pattern [20], both being a consequence of the formation of various forms of bromelain isolated from crude bromelain [21]. Furthermore, different purification methods and several purification steps could also contribute to molecular weight heterogeneity. The optimum temperature range for stem bromelain is between 40 and 60 °C (37–70 °C for fruit bromelain) and its optimum pH range is 4–8 (3–8 for fruit bromelain) [1,13,15,22,23,24]. However, its activity is no longer susceptible to the effect of the pH once it is combined with a substrate [1]. Bromelain preferentially cleaves glycyl, alanyl and leucyl peptide bonds [25]. Its activity can be determined using different substrates, including casein [16,26,27,28], gelatin [1], azocasein [19,29], azoalbumin, hemoglobin, sodium caseinate [23,30], and synthetic peptide substrates (Nα-CBZ-ι-Lysine p- nitrophenyl ester, Z-Arg-Arg-pNa, Bz-Phe-Val-Arg-pNA, H-Val-Ala-pNA, Suc-Ala-Ala-Val-pNA, Suc-Ala-Pro-Leu-Phe-pNA, Suc-Phe-Leu-Phe-pNA, Z-Phe-Arg-pNA and Z-Phe-pNA) [31,32]. The value of Michaelis–Menten constant (K_m_) vary significantly when different substrates (azoalbumin, azocasein, sodium caseinate, casein and hemoglobin) are used for fruit bromelain activity determination, being the lowest (0.026 mM) for azoalbumin and the highest (0.165 mM) for hemoglobin [33]. The most suitable substrate for the fruit bromelain activity is azocasein, followed by azoalbumin, casein, sodium caseinate and hemoglobin according to the enzyme catalytic power parameter (V_max_/K_m_ ratio), being 0.104, 0.096, 0.022, 0.020 and 0.014, respectively [33]. Bromelain inactivation rate follows first-order kinetics at 55 °C and 60 °C, but not above 70 °C, while its thermal deactivation is entirely irreversible and follows a two-stage mechanism, including the formulation of an intermediate between native and denatured states [15]. Bromelain retains more than 50% of its original proteolytic activity after 30 min incubation at 60 °C, from 9% to 22% after 15 min incubation at 70 °C, and becomes utterly inactive when heated for 10 min at 100 °C [34]. Aqueous proteolytic activity of bromelain decreases rapidly at 21 °C, while its concentrated forms (>50 mg/mL) are stable for one week at room temperature and can be repeatedly frozen and thawed [35].

**Table 1:** Physiochemical properties of cysteine endopeptidases derived from pineapple plants [1,13,15,22,23,24].

	Stem Bromelain	Fruit Bromelain	Ananain	Comosain
Source	Pineapple stem	Pineapple fruit	Pineapple stem	Pineapple stem
Molecular weight [kDa]	23.8–37.0	23.0–32.5	23.4–25.0	24.4–24.5
Isoelectric point	≥9.5	4.6	>10	>10
Amino acid sequence	212, 291, 285	326, 351	216	186
Optimum T [°C]	40–60	37–70	/	/
Optimum pH	4–8	3–8	/	/
Presence of Glycoproteins	Yes	Yes/No	No	Yes

The activation energy of bromelain is 41.7 kcal/mol [23], and same can be activated by many chemical agents, including calcium chloride, cysteine, sodium cyanide, bisulfate salt, hydrogen sulfide, sodium sulfide and benzoate [13,36,37]. Stem bromelain is reversibly inhibited during reaction with organic mercury, ions of mercury and tetrathionate. Its irreversible inhibition occurs by reacting with *N*-ethylmaleimide, *N*-(4-dimethyl-3,5-dinitrophenyl) maleimide, monoiodoacetic acid and 1,3-dibromine acetone due to alkylation of the thiol group, an essential group for the activity of the enzyme [15].

**Figure 1:**
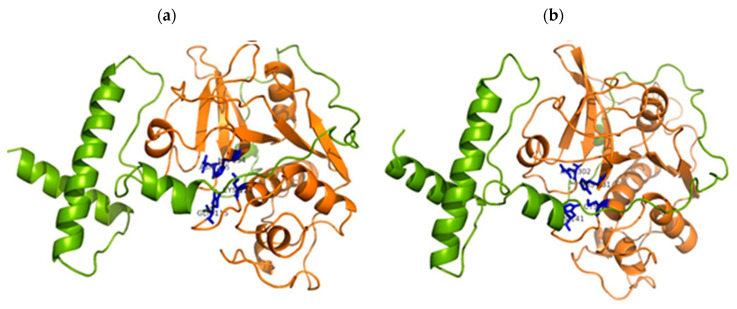
Model domain organisation of (**a**) fruit bromelain (sequences with the accession number of BAA21848 in the NCBI Genbank database and 352 amino acids) and (**b**) stem bromelain (sequences with the accession number of CAA08861 in the NCBI Genbank database and 357 amino acids). α-helix domain (domain I29 at the N-terminal region) is colored in green, β-sheet domain (domain peptidase C1 at the C-terminal region) is colored in orange. The catalytic amino acids of both models are represented as sticks (Reproduced with permission from [13], Elsevier, Amsterdam, The Netherlands, 2018).

Until now, several different (fruits or stem) bromelain amino acids sequences have been deposited in the National Center for Biotechnology Information (NCBI) Genbank database with around 90–100% similarity. Alanine, glycine and serine are the most abundant amino acids in stem and fruit bromelains, while histidine is present in the lowest amount [13]. Bromelain amino acid sequence is highly similar to papain, actinidin, proteinase Ω and chymopapain [24]. A single polypeptide chain constitutes the primary structure of bromelain with amino acids folded into two structure domains: α-helix domain (domain cathepsin propeptide inhibitor—I29) and antiparallel β-sheet domain (domain peptidase C1) (Figure 1). Mainly, the I29 domains are located between amino acids number 1 and 100 of the N-terminal sites. The structure domains are stabilized by disulfide bridges and numerous hydrogen bonds. Stem bromelain differs from the fruit bromelain in the number of polar amino acids (arginine and lysine), and acidic amino acids (aspartate and glutamate). The stem bromelain contains more polar amino acids, and the fruit bromelain has more acidic amino acids, leading to a difference in isoelectric point (4.6 and ≥9.5 for fruit and stem bromelain, respectively). The active site is located on the surface molecules between domains and the proposed catalytic residues for the modeled BAA21848 structure is composed of three amino acids Cys-121, His-254 and Asn-275; for CAA08861 structure Cys-147, His-281 and Asn-302 are proposed, which fall into approximately the exact locations as in papain catalytic residues (Cys-25, His-159 and Asn-175) [13,38].

### 2.2. Isolation, Extraction and Purification

Bromelain can be isolated from all parts of the pineapple plant (stem, core, peel, crown and leaves), which affect the concentration and composition. The stem and pineapple fruit allow the production of high amounts of bromelain, while the pineapple core, peel and leaves contain smaller quantities, yet, together with pineapple stem and crown, they represent up to 50% (*w/w*) of the total pineapple waste [16], making extraction of bromelain from pineapple waste economically and environmental attractive [28]. Consequently, the most commercially available bromelain is usually obtained from pineapple stem, which is also therapeutically more effective and shows higher proteolytic activity than fruit bromelain [17].

**Figure 2:**
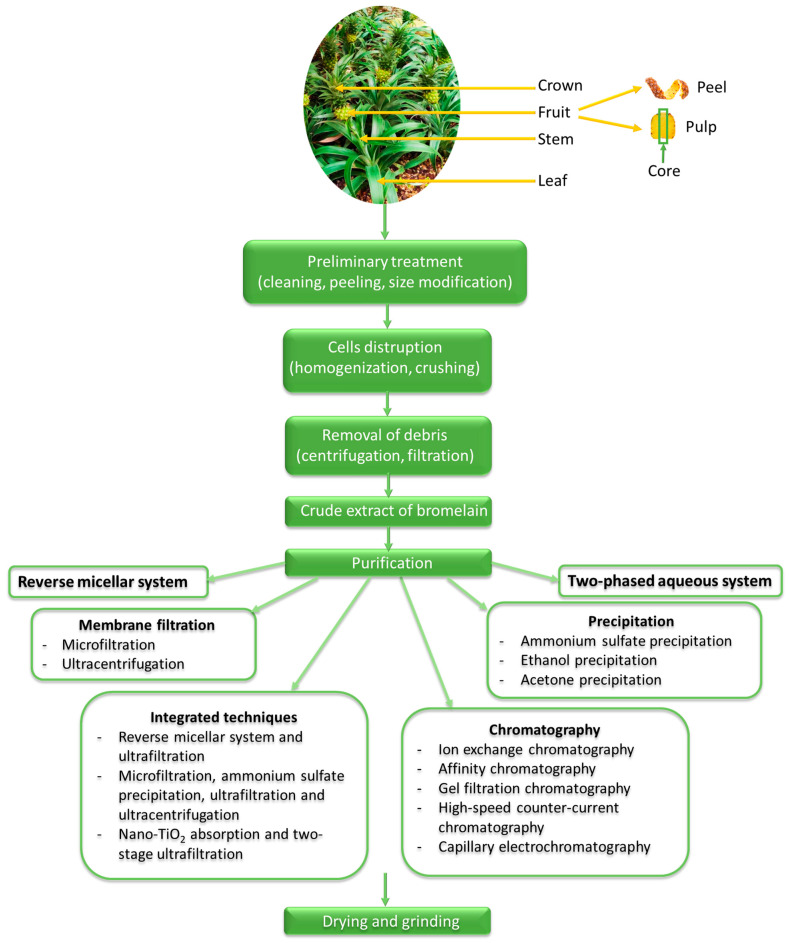
Scheme of a typical bromelain production.

Numerous strategies have been developed for the extraction and purification of bromelain. The bromelain production process consists of several sequential steps, as depicted in Figure 2. Fresh pineapple stem parts or any other parts of the pineapple are washed, cut into small pieces, crushed in an industrial blender to disrupt the plant cells and separate the enzyme from the cells, filtered to remove the fibrous material and centrifugated to remove insoluble materials [1,38,39,40]. The obtained supernatant is called crude extract and is further purified as impurities and by-products (e.g., proteins, pigments, polysaccharides) can react with bromelain and inhibit its activity [17]. Purification can be done using chromatographic processes (among them ion-exchange chromatography with prior precipitation by adding ammonium sulfate is the most relevant), a two-phase aqueous system (e.g., PEG/K_2_SO_4_, PEG/MgSO_4_, PEG/poly(acrylic acid), PEG/(NH_4_)_2_SO_4_) or a reverse micellar system [1,17,41], the selection primarily depends on the application. Purification can also be performed by membrane-based processes (microfiltration, ultrafiltration) [40] or precipitation, followed by centrifugation and solubilization in phosphate buffer [38]. The residual specific activity of crude pineapple extract purified by fractionation using ammonium sulfate at 20–50% saturation level is 70 U/mg with the total activity of 167.3 U, total protein content of 2.39 mg and the purity level of 5.3 fold compared to the crude enzyme extract [16]. When acetone (50–80% saturation) was used as fractionating agent, the residual specific activity of bromelain fraction was 19.7 U/mg [16]. The crude bromelain of pineapple fruit purified by high-speed counter-current chromatography coupled with the reverse micelle solvent system yielded 3.01 g of bromelain from 5.00 g crude extract in 200 min [42]. The choice of a purification method determines the purity of the enzyme and the enzyme production cost [40]. Commercially available bromelain is produced by a lengthy and costly purification method that yields bromelain in varying degrees of purity [32]. The purification steps correspond to 70–90% of the total production costs [38], implying the need to develop innovative, cost-efficient methods for pure bromelain production in fewer steps [39].

Isolation of bromelain from pineapple fruit and its various parts is not the only way to obtain bromelain; researchers are also trying to clone the bromelain gene in multiple hosts, such as *E. coli* BL21-AI [32,43,44], *E. coli* BL21-CodonPlus(DE3) [45], *E. coli* BL21 DE3pLysS [14], *Pichia pastori* [46] and Chinese cabbage (*Brassica rapa*) [47], leading to recombinant bromelain—an intracellular enzyme abundant in the cytoplasm of the host cell, meaning that the host cell wall needs to be disrupted using homogenization, chemical lysis, sonication with lysozyme or freeze-thawing to release the bromelain [44]. Amid et al. [32] reported about higher specific activity of recombinant bromelain (1.231 U/mg) in comparison to commercial bromelain (0.846 U/mg) when the release of p-nitrophenol from a synthetic substrate Nα-CBZ-ι-Lysine p-nitrophenyl ester was monitored. The recombinant bromelain obtained in a single step immobilized metal affinity chromatography was purified 41-fold and showed optimum activity at pH 4.6 and 45 °C [32]. In contrast, George and co-workers [14] reported a higher protease activity of native bromelain obtained from Sigma (a purified form of crude stem bromelain) in comparison to recombinant bromelain when casein was used as a substrate. Crude bromelain showed even higher proteolytic activity than native bromelain due to its composition of a mixture of protease complexes which can cleave substrate even more effectively. However, the effectiveness of the extraction of the (recombinant) bromelain and its residual activity are related to the choice of buffer, presence of chelating agents (ethylenediaminetetraacetic acid (EDTA), cyclohexane-1,2-diaminoetetraacetic acid (CDTA), hydroxyethyl ethylenediamine triacetic acid (HEDTA)), reducing agents and protease inhibitors [44].

### 2.3. Bioactivity

**Figure 3:**
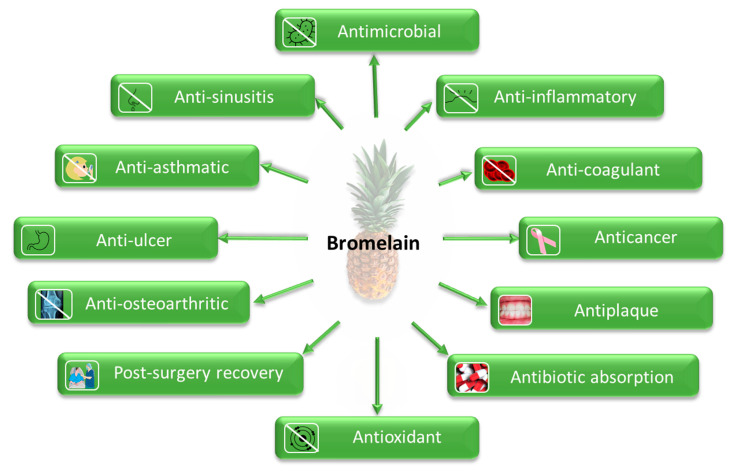
Overview of the bromelain bioactivity.

Bromelain has been a valuable compound in traditional medicine in Southeast Asia, Kenya, India, and China for a long time [28,48] due to its numerous therapeutic effects (Figure 3), including antimicrobial [16,49,50], anti-inflammatory [30,51], anticoagulant [52], anticancer [53,54], antiplaque [55,56], and antiulcer properties [50]. Furthermore, it is also beneficial for wound healing [57,58,59,60], dermatological disorders [19], post-surgery recovery, enhanced antibiotic absorption [1], treatment of osteoarthritis [61], sinusitis and diarrhea [17]. Recently, bromelain is suggested as an antiviral agent against COVID-19 due to the inhibition of different versions of SARS-CoV-2 [62]. Some of its therapeutic mechanisms are discussed below.

The mechanism behind the antimicrobial activity of bromelain is not well known, yet, is believed that bromelain may hinder bacterial growth by hydrolyzing some peptide bonds in the bacterial cell wall [14]. When bromelain digests the surface proteins, the cell wall is damaged, allowing the cell to leak, swell, and open [1]. Bromelain also inhibits the growth of some bacteria by preventing bacterial adhesion to specific glycoprotein receptors on the surface [1,48]. Furthermore, bromelain inhibits enterotoxin production of *Escherichia coli* (*E. coli*) and prevents diarrhea caused by *E. coli* [17]. Bromelain shows antimicrobial activity against both Gram-positive and Gram-negative bacteria, including *E. coli*, *Aggregatibacter actinomycetemcomitans* (*A. actinomycetemcomitans*), *Porphyromonas gingivalis* (*P. gingvalis*), *Streptococcus mutans* (*S. mutans*) [56], *Bacillus subtilis* (*B. subtilus*), (*S. aureus*), *Pseudomonas aeruginosa* (*P. aeruginosa*), *Proteus* spp., *Acinetobacter* spp., … [1,63]. Additionally, synergistic use of bromelain and antibiotics increases the antibacterial effect due to increased absorption of antibiotics induced by bromelain, leading to better drug distribution in the microbes [1,17]. Bromelain has also been reported to act as an inhibitor of fungal pathogens [39,64].

**Figure 4:**
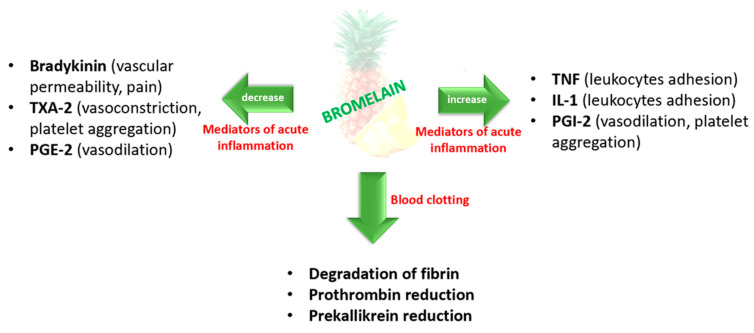
Anti-inflammatory and anticoagulant mechanisms of action of bromelain (Adapted with permission from [65], MDPI, Basel, Switzerland, 2021).

Inflammation is the body’s attempt to protect itself [28]. It is a complex biological mechanism primarily regulated by the disruption of tissue homeostasis [17]. Most often, non-steroidal anti-inflammatory drugs are prescribed to combat the classic signs of inflammation (heat, pain, redness and swelling), leading to severe damage to the gastrointestinal tract and numerous side effects. In such cases, the bromelain can be used as an alternative [28] due to its anti-inflammatory activity mediated by (Figure 4):

- increased serum fibrinolytic activity, reduced plasma fibrinogen levels and decreased bradykinin levels (resulting in reduced vascular permeability), thereby reducing edema and pain;
- modulating the formation of pro-inflammatory prostaglandins (by lowering levels of prostaglandin E2 (PGE2) and thromboxane A2 (TXA-2)), enhancing the anti-inflammatory mediators and the levels of prostaglandin I2 (PGI-2);
- modulating specific immune cell surface adhesion molecules—acting on the migration of neutrophils to inflammation sites [28,61,65].

Because of these actions, bromelain is potentially effective in several conditions and diseases associated with inflammation, including rheumatoid arthritis, osteoarthritis, cardiovascular diseases, skin wounds and burns, perioperative sports injuries and chronic rhinosinusitis [65]. Furthermore, inflammation is also associated with cancer; suppressing chronic inflammation may inhibit cancer progression due to reduced PGE-2 and prostaglandin-endoperoxide synthase 2 (COX-2) after bromelain administration [17]. The anti-inflammatory effect of bromelain is also the most traditional and established one [17].

Bromelain affects blood clotting by increasing the fibrinolytic capacity of serum and inhibiting the synthesis of the blood-clotting protein fibrin (Figure 4). It also decreases prekallikrein—a proenzyme that must be converted to kallikrein to help in coagulation. Consequently, it inhibits the generation of bradykinin, leading to pain and edema reduction, and increased circulation on the side of the injury [17,28,39].

The molecular mechanisms of bromelain’s anticancer activity are also not fully understood [11]. However, some research has suggested that the bromelain anticancer mechanism is mainly attributed to its protease components and proteolysis [11,35]. One of the described anti-tumor mechanisms of bromelain includes induced differentiation of leukemic cells, leading to apoptosis of tumor cells [1]. Bromelain inhibits the growth of cancer cells by increasing the expression of two activators of apoptosis in mouse skin—p53 and Bax [66]. It also decreases the activity of cell survival regulators such as Akt and Erk, promoting apoptotic cell death in tumors. Expression of promoters of cancer progression—nuclear factor kappa B (NF-κB) and Cox-2 are also inhibited by bromelain in mouse papillomas and models of skin tumorigenesis [1,11].

Bromelain is well tolerated and considered a safe nutraceutical with no serious adverse effects [30,65]. It has already received FDA approval for clinical use as an orally administered anti-inflammatory and anticoagulant therapeutic [52]. Its oral administration is well tolerated even in high doses (up to 3 g/day) for prolonged therapy periods, even up to several years [11]. It has a very low level of toxicity [48]. The lethal dose (LD50) for intraperitoneal administration is 37 mg/kg and 85 mg/kg for mice and rabbits, respectively, and 30 mg/kg and 20 mg/kg for intravenous administration [65], with no immediate toxic reactions [25]. Daily oral administration of 500 mg/kg of bromelain did not provoke any alteration in food intake, growth, histology of the heart, kidney and spleen, or hematological parameters in rats [25]. After daily bromelain administration up to 750 mg/kg no toxicity was observed in dogs after 6 months [17]. No relevant side effects have been observed in humans at doses of up to 2000 mg/kg, even with prolonged oral administration [65]. However, clinical trials have reported some side effects, mainly gastrointestinal (i.e., diarrhea, nausea and flatulence), headache, tiredness, dry mouth, allergic reactions, and bleeding risk, especially in individuals treated with other anticoagulant drugs [17,61,65].

### 2.4. Immobilization Strategies

**Figure 5:**
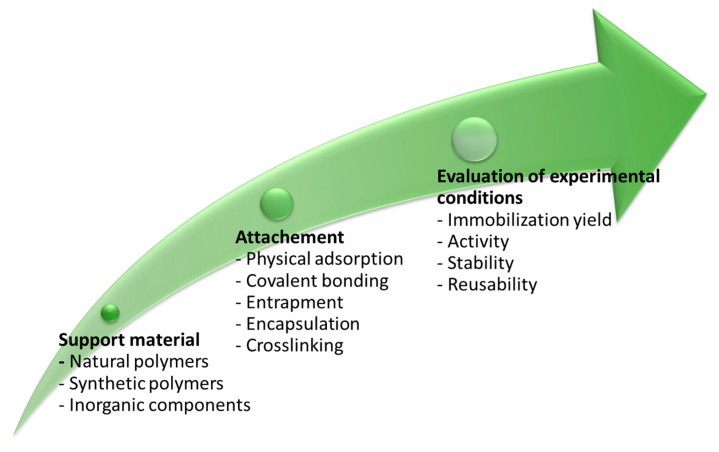
Steps for immobilization of bioactive enzymes.

One of the issues related to enzymes (such as bromelain) utilization is a decline of their activity with time or after processing. Indeed, enzymes, isolated from their natural environments, are susceptible to process conditions, such as pH, temperature, strong acids and bases, and non-aqueous solvents, which may affect their activity [67], health benefits and pharmaceutical applicability [68]. A promising strategy to secure their efficiency is immobilization [69], which requires selecting supporting material (inorganic components, synthetic polymers or natural polymers) with suitable surface chemistry for controlled enzymatic attachment. The next step is optimizing the immobilization process towards desired immobilization yield, activity retention of even amplification, stability and reusability [69] (Figure 5). Successful immobilization requires thorough knowledge and control of the interactions between the carrier and the enzyme [70]. The choice of immobilization method and carrier depends on the nature of the immobilized compound and the goal of immobilization (resistance against high temperature, pH, controlling the release, preventing negative interactions…) [71].

**Figure 6:**
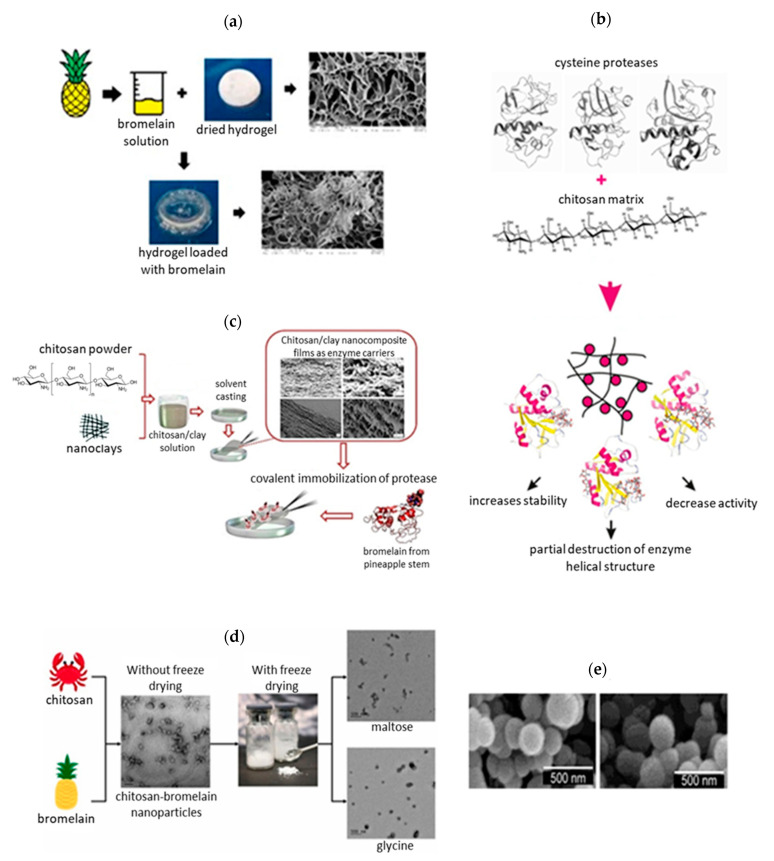
Schematic illustration and SEM micrographs of immobilization methods of bromelain: (**a**) entrapment into hydrogels (Reproduced with permission from [86], Elsevier, Amsterdam, The Netherlands, 2018); (**b**) adsorption onto chitosan matrix (Reproduced with permission from [70], Elsevier, Amsterdam, The Netherlands, 2021); (**c**) covalent immobilization (Reproduced with permission from [81], Elsevier, Amsterdam, The Netherlands, 2018); (**d**) entrapment into nanoparticles (Reproduced with permission from [29], Elsevier, Amsterdam, The Netherlands, 2021); (**e**) SEM micrographs of encapsulated silica nanoparticles formed without bromelain (left) and with bromelain (right) (Reproduced with permission from [72], John Wiley and Sons, Hoboken, NJ, USA, 2014).

Immobilization methods and carriers utilized for immobilization of bromelain in the last 10 years are summarized in Table 2 and Figure 6. Bromelain has been combined mostly with nanoparticles, hydrogels, fibers and matrices with the aim to improve the properties of the final formulation [29]. Baker and co-workers [72] encapsulated bromelain in silica nanosphere aggregates, using sodium metasilicate as a silica precursor and ethyleneamines (diethylenetriamine (DETA), triethylenetetramine (TETA), tetraethylenepentamine (TEPA), and pentaethylenehexamine (PEHA)) of different chain lengths as initiators. They found out that increased loading mass of bromelain resulted in the increased activity of bromelain, being 61.7% when 10 mg of bromelain was encapsulated in silica and only 12.1% when 2 mg of bromelain was used. The encapsulation also increases the thermostability with maximum activity at 40 °C for free bromelain and at 50 °C for encapsulated bromelain. At 70 °C free bromelain lost its activity while encapsulated bromelain retained approximately 30% of its activity [72]. Chitosan-methyl cellulose hydrogel [73], freeze-dried chitosan nanoparticles [29], chitosan microspheres [74], poly(lactide-co-glycolic) acid nanoparticles [75] and katira gum nanoparticles [76] have also been studied for encapsulation of bromelain, showing various immobilization yield and bromelain activity. Esti et al. [77] covalently immobilized stem bromelain on chitosan beads by direct mechanism, involving the bromelain carboxyl groups of Asp or Glu residues and the amino groups of the chitosan. Ataied et al. [78] studied bacterial nanocellulose as a support material for physical adsorption of bromelain and reported about 9-times increased antimicrobial activity of adsorbed bromelain. Holyavka et al. [70] also used the adsorption method for immobilization of cysteine proteases onto chitosan and observed significant loss of the bromelain catalytic activity due to: (a) nonspecific binding, (b) structural changes of bromelain upon interaction with the carrier, and (c) diffusional and steric limitations, leading to impeded access of the active bromelain center [70]. All the studies clearly show the influence of the carrier and immobilization method on bromelain’s immobilization yield, residual activity, and thermal stability. By choosing a suitable carrier and immobilization method, it is possible to significantly reduce the influence of the carrier on the structural and functional properties of the bromelain [70], enhance its stability and activity upon exposure to a wide range of pH and high temperatures and improve its antimicrobial and anti-inflammatory activity. However, there is not yet a standard, highly efficient immobilization approach for bromelain delivery [29].

**Table 2:** Review of bromelain immobilization methods.

Immobilization Method	Carrier/Support Material	Crosslinking Agent or Initiator	Outcomes	References
Covalent immobilization	APTES– modified mesoporous silica nanoparticles (MSN)	1-ethyl-(3-dimethylaminopropyl) carbodiimide/N-hydroxysuccinimide (EDC/NHS)	- Enhanced diffusion of MSN within the tumor extracellular matrix	[52]
	Chitosan beads (Chitopearl BCW-3010)	/	- 22% immobilization yield - Higher resistance to the SO_2_, skin and seed tannins	[77]
	Lyocell fibres	Epichlorohydrin + glutaraldehyde (GA), EDC, and APTES + GA	- 88.14% activity yield of immobilized bromelain at pH 7 - High stability of immobilized bromelain at pH range 6–8 - pH 7 is ideal for immobilization	[79]
	Chitosan–cobalt–magnetite nanoparticle	GA	- 77% immobilization binding - 85 ± 2% of the initial catalytic activity retained - 50% of the initial catalytic activity after the fifth use	[80]
	Chitosan—clay (montmorillonites/bentonites or sepiolite) nanocompositefilm	GA	- Increased immobilization yield, decreased catalytic activity of the immobilized bromelain	[81]
Adsorption	Chitosan matrix	/	- Increased stability of bromelain concerning UV irradiation in comparison with free enzymes - Chitosan matrix acts as photoprotector	[82]
	Chitosan colloidal particles	/	- Destruction of a part of the helical structure - Decreased catalytic activity of bromelain	[70]
	Magnetic carbon nanotubes	/	- Adsorption followed second-order kinetics - Bromelain (c = 100 μg/mL) alone and in combination with nanotubes efficiently inhibited the HT-29 colorectal cancerous cells	[66]
	Ag nanoparticles	/	- Spontaneous interaction of AgNP with bromelain - Main forces are electrostatic and hydrophobic interactions - Adsorption follows pseudo-second-order kinetics	[83]
	Magnetic nanoparticles with chitosan and reactive red 120 (Red 120-CS-MNP)	/	- Red 120-CS-MNP are suitable carrier - Adsorption isotherm fitted the Freundlich model well	[84]
	Spores of the probiotic *Bacillus*	/	- Improved stability and activity of the bromelain upon exposure to a wide range of pH and high temperatures	[85]
	Bacterial nanocellulose	/	- Improved antimicrobial activity	[78]
Entrapment	N-isopropylacrylamide (PNIPAAm) hydrogels	/	- New release system evolving hydrogels and bromelain for wound healing	[86]
	Alginate—arabic gum hydrogels	/	- 19% of bromelain was incorporated, 227% swelling ratio of final hydrogel	[87]
Encapsulation	Silica nanoparticles	DETA, TETA, TEPA, or PEHA	- Increased thermal stability	[72]
	Chitosan—methyl cellulose hydrogel	GA	- Bromelain as a drug for digestion problem	[73]
	Freeze-dried chitosan nanoparticles	Sodium tripolyphosphate	- 85.1 ± 1% encapsulation efficiency - Chitosan-bromelain-nanoparticles presented 4.9 U/mL of enzymatic activity (104.7% of free bromelain activity) - Freeze-dried chitosan-bromelain-nanoparticles improve bromelain and nanoparticle stability (maltose as lyoprotectant)	[29]
	Katira gum nanoparticles	/	- Enhanced anti-inflammatory activity of bromelain against carrageenan	[76]
	Glutaraldehyde crosslinked chitosan microspheres	/	- 84.75% encapsulation efficiency	[74]
	Poly(lactide-co-glycolic) acid nanoparticles	/	- 48 ± 4.81% entrapment efficiency - Enhanced antitumor effect	[75]
	Poly(lactide-co-glycolic) acid nanoparticles	/	- Oral administration of encapsulated nanoparticles reduced the tumor burden of Ehrlich ascites carcinoma in mice and increased their life-span (160.0 ± 5.8%) when compared with free bromelain (24 ± 3.2%) - Enhanced anti-carcinogenic potential upon oral administration	[53]
	Eudragit L 100 nanoparticles	/	- 85.42 ± 5.34% entrapment efficiency - Lyophilized formulation ensured 2-year shelf-life at room temperature - Oral bromelain delivery in inflammatory conditions	[88]
	Nanostructured lipid carrier (lecithin-steric acid-Span-80) emulsified with PVA solution		- ~77% entrapment efficiency - Diminished of paw edema, joint stiffness, mechanical allodynia, tissue damage - Alleviation of oxidative stress and immunological markers - Application in rheumatoid arthritis	[89]

### 2.5. Applications

Bromelain finds widespread applications in several areas, including medicine, health, food, and cosmetics [15]. In the food industry, it is used for meat tenderization [90,91,92] (together with papain representing 95% of the enzymes used to tenderize meat in the USA [32]), baking process [93], protein hydrolysate production [94], as a food supplement [95,96,97] and as an anti-browning agent in fruit juices [98]. Furthermore, bromelain also shows antimicrobial activity against *Alicyclobacillus acidoterrestris* (*A. acidoterrestris*), Gram-positive bacteria often related to the deterioration of acidic products (citrus juices, iced tea, isotonic drinks and tomato extract) [99]. Still, its main application continues to be in the pharmaceutical industry [24].

**Figure 7:**
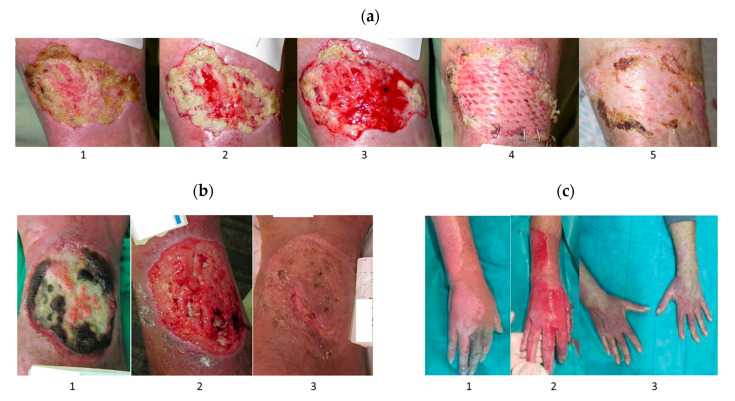
Bromelain-based treatment (BBT): (**a**) venous insufficiency ulcer; 1—pre-existing for 5 months, 2—after first BBT 4-h application, 3—after fourth BBT 4-h application (16 h total exposure to bromelain-based debridement), 4—one week post-split-thickness skin grafting, 5—seven weeks post-split-thickness skin grafting (Adapted with permission from [108], John Wiley and Sons, Hoboken, NJ, USA, 2018); (**b**) large venous leg ulcers; 1—venous leg ulcer pre-existing 10 weeks, 2—after 7 BBT, and 3—two months after split-thickness skin grafting (Reproduced with permission from [107], John Wiley and Sons, Hoboken, NJ, USA, 2021); (**c**) hand burn; 1—before BBT, 2—after BBT, 3—outcome 38 days post-burn (Reproduced with permission from [104], Baoshideng Publishing Group Inc., Pleasanton, CA, USA, 2017).

Several experimental data and clinical studies showed better burns and wound healing under the influence of bromelain due to its proteolytic, anti-inflammatory, antibacterial, and anti-edematogenic effects [58,59,73,86,100,101,102]. Recently, Chen et al. demonstrated reduced inflammation and improved wound healing rate in a rat model when treated with bromelain-immobilized electrospun poly(ε-caprolactone) fibres [100]. These fibres also effectively prevented wound infections due to their antibacterial activity against Gram-positive bacteria *S. aureus*, dominant in the initial stage of chronic wound formation, and Gram-negative bacteria *E. coli* [100]. Aichele et al. confirmed the effect of bromelain on myofibroblast reduction, resulting in attenuated fibrotic development [58]. Topical application of bromelain is effective in the eschar removal (debridement) of uncomplicated gunshot wounds when used as an adjunct to a simple wound incision and simplifies the conventional wound excision treatment [103]. Bromelain treatment has a characteristic of attacking mainly necrotic tissue, while healthy tissue seems unaffected [58]. One example is bromelain-based enzymatic debridement product NexoBrid (produced by MediWound Ltd., Yavne, Israel), which reduced infection, blood loss, length of hospital stays, and the need for skin grafting in treating deep partial and full-thickness burns due to early non-surgical eschar removal without harming surrounding viable tissue (Figure 7) [59,101,104,105]. The NexoBrid, a topically-applied concentrate of proteolytic enzymes enriched in bromelain, was clinically approved in 2012 by the European Medicines Agency (EMA) to remove dead tissue in severe skin burns, and until now is the only clinical-approved application of bromelain [106]. Moreover, EscharEx (MediWound Ltd., Yavne, Israel) is another bromelain-based enzymatic debridement currently in development for chronic wounds [107,108]. Several researchers have also incorporated bromelain into various hydrogels [73,86,87,102] to create a dressing that ensures a moist environment around the wound and provides a barrier against infection [87].

Bromelain has clinical potential for the treatment of skin problems such as acne owing to its antimicrobial activity against microbial flora that is often associated with acne infection, including *P. acne*, *S. aureus*, *C. diphtheria* and *E. coli*, among which *S. aureus* was the most susceptible organism to the action of bromelain extracts, followed by *P. acne* [16,19].

**Figure 8:**
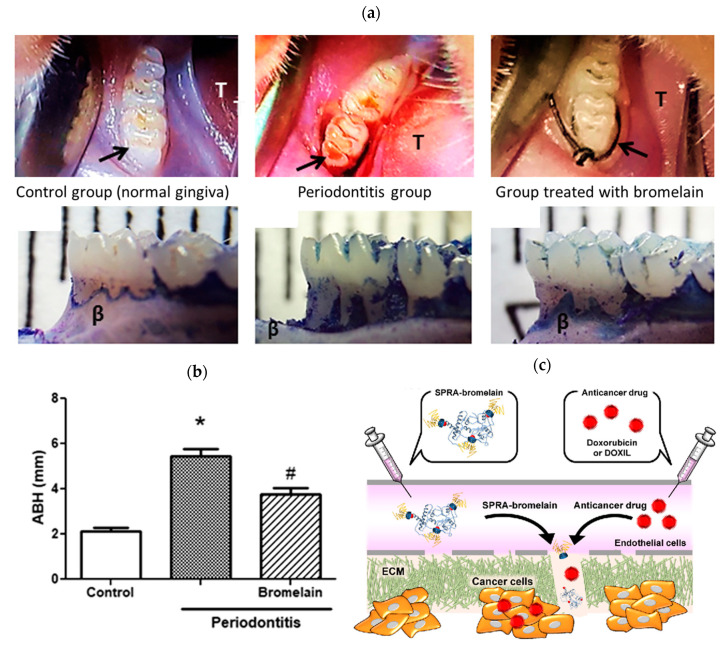
(**a**) Comparison of the control group (normal gingiva), periodontitis group and group treated with bromelain 15 mg/kg (arrow shows the first molar and the letter T shows the tongue). Group treated with bromelain indicates improvement of gingival papilla staining, reduction in edema, absence of bleeding and moderate bone loss (Reproduced with permission from [111], John Wiley and Sons, Hoboken, NJ, USA, 2020); (**b**) morphometric analyses of alveolar bone height; * *p* < 0.05 indicates the Periodontitis groups versus the Control group and # *p* < 0.05 indicates the Periodontitis groups versus the Bromelain group (Reproduced with permission from [111], John Wiley and Sons, Hoboken, NJ, USA, 2020); (**c**) the scheme of the SPRA-bromelain suggested a mechanism of ECM-degradation in pancreatic cancer (Reproduced with permission from [121], ACS Publications, Washington, DC, USA, 2020).

In addition, bromelain can be used to inhibit the growth of bacteria that causes dental caries due to the intense antimicrobial activity against *P. gingvalis* (diameter of clear zone of 21 mm) [56]. The minimum inhibitory concentration (MIC) of bromelain against microorganisms associated with periodontal diseases was also determined by Praveen and co-workers, being 2 mg/mL, 4.15 mg/mL, 16.6 mg/mL and 31.25 mg/mL for *S. mutans*, *P. gingivalis*, *A. actinomycetemcomitans* and *Enterococcus fecalis* (*E. fecalis*), respectively [50]. The minimum bactericidal concentration (MBC) of crude bromelain of pineapple fruit to multidrug-resistance Gram-negative *P. aeruginosa* is 0.75 g/mL [109]. *P. aeruginosa* is a leading cause of nosocomial infections, responsible for 10% of hospital-acquired infections [109]. Crude bromelain, extracted from pineapple fruit, exhibited a 12 mm zone of inhibition against *Streptococcus pneumoniae* (*S. pneumoniae*), *P. aeruginosa* and *S. aureus* at a concentration of 1.0 g/mL [63]. Crude bromelain extracted from pineapple crown leaf (aqueous extract of pineapple crown leaf) showed 70–95% inhibition of microbial growth with MIC range of 1.65–4.95 mg/mL against laboratory strain *Saccharomyces cerevisiae* (*S. cerevisiae*) and *E. coli* XL1 blue, type strain *S. aureus*, drug-resistant strain *E. coli* DH5α pet16b Amp^r^ and two pathogenic strain *B. subtilis* and *Candida albicans* (C. *albicans*) [18]. It is also hypothesized that bromelain inhibits the development and progression of periodontitis through the elimination of important cell surface molecules (CD25) in leucocytes (proteolytic activity of bromelain), decreased growth of periodontal microorganisms (anti-adhesion property), reduced migration of neutrophils to periodontal sites (the hyperactivity of the neutrophils leads to damage of the periodontium), downregulating of inflammatory mediators (COX-2, tumor necrosis factor (TNF)), decreased osteoclastogenesis process with reduction in alveolar bone loss (Figure 8a,b) [110,111]. A clinical study conducted by Odresi et al. confirmed the anti-edematous action of bromelain in third molar surgery. The group treated with bromelain showed a reduced inflammatory response compared to the control group [112].

The anticarcinogenic effect of bromelain has been investigated through in vitro studies involving various cancer cell lines [66]. It can inhibit the growth and proliferation of mouse breast carcinoma 4T1, human breast adenocarcinoma GI-101A and MCF7, human prostate carcinoma PC3 and human gastric carcinoma AGS in a dose-dependent manner [43,113,114,115]. Bromelain concentration >75 µg/mL remarkably decreased cell viability in MCF7, PC3 and AGS human cell lines as a single therapy [113]. Moreover, it is also effective as an anticancer agent against cell lines of melanoma (A375), epidermoid carcinoma (A431) [116], gastric carcinoma (KATO-III and MKN45) [117], colorectal cancer (human colon adenocarcinoma (Caco-2)) [118], ovarian cancer (A2780), colon cancer (HT29) [119], lung cancer [120], pancreatic [121] and liver cancer (hepatocellular carcinoma HepG2) [10]. The absorption and efficiency of chemotherapy drugs (5-fluorouracil, vincristine, cisplatin, idarubicin, doxorubicin), antibiotics (amoxicillin and tetracycline) or blood pressure medication (captopril and lisinopril) [17,122,123,124] can be potentiated when combined with oral, subcutaneous or intramuscular administration of bromelain [17]. Higashi et al. [121] investigated whether bromelain could be used to degrade the barrier of dense extracellular matrix (ECM), a characteristic inhibitor of penetration of anticancer drugs in the treatment of pancreatic cancer. Due to the short half-life of the bromelain in the blood, they prepared reversibly PEGylated bromelain using “self-assembly PEGylation retaining activity (SPRA)” technology, thus retaining high bromelain activity and causing ECM degradation and increase of anticancer drugs in tumor tissue of pancreatic cancer (Figure 8c) [121]. Encapsulated bromelain also enables slow delivery, thus being favorable for cancer treatment [66].

Bromelain effectively reduces the risk of clots-associated problems, including stroke or heart attack [15,17,25] due to the breaking down of the blood-clotting protein fibrin [125]. Bromelain has been shown to be effective in treating rheumatoid arthritis [86], exercise-induced muscle injuries [125] and edema caused by post-surgical trauma [19]. It was also used in treating patients with osteoarthritis, where it worked similarly to diclofenac treatments [126]. In combination with *Boswellia serrata* (*B. serrata*)*,* bromelain improved the quality of life of patients suffering from different forms of osteoarthritis [96].

## 3. Nisin

### 3.1. Structural and Biological Properties

**Figure 9:**
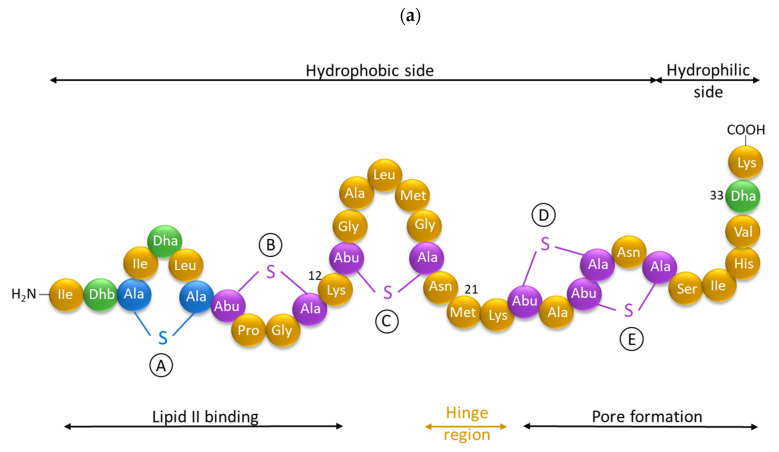

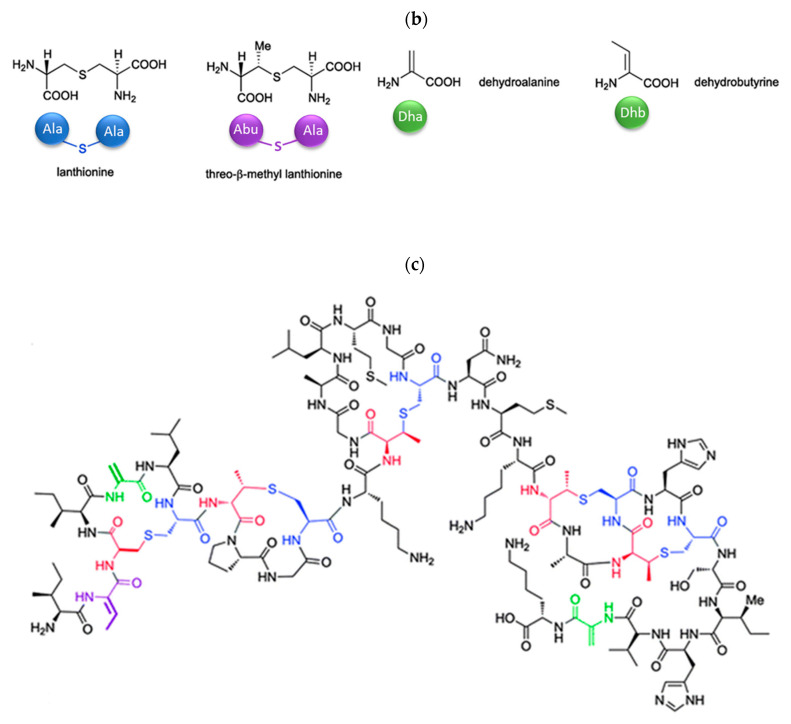
(**a**) Primary structure of nisin Z with highlighted residues involved in crucial aspects of the antimicrobial activity (Adapted with permission from [145], Elsevier, Amsterdam, The Netherlands, 2018); (**b**) chemical formula of dehydroalanine (Dha), dehydrobutyrine (Dhb), lanthionine (Lan), and β-methyl lanthionine (MeLan) (Adapted with permission from [145], Elsevier, Amsterdam, The Netherlands, 2018); (**c**) chemical structure of nisin A (Reproduced with permission from [146], RSC, Cambridge, UK, 2012).

Antimicrobial peptides (AMPs) are cationic, hydrophobic or amphipathic natural antibiotics, consisting of amino acid residues of varying lengths (up to 100) in a linear or cyclic arrangement [127], derived from bacteria, insects, plants, birds, amphibians, fish, and mammals [128,129,130]. AMPs have attracted much attention because of their potent antibacterial activity against a broad spectrum of microorganisms, multiple modes of action, a low bacterial resistance rate, ability to destroy target cells rapidly and low cytotoxicity [127,131,132,133,134], therefore showing potential to overcome the growing problems of antibiotic resistance [135,136]. An example of AMPs is also an odorless, colorless, tasteless substance—nisin [131]. It is a cationic, amphiphilic, antimicrobial polypeptide [137,138], ribosomally synthesized and posttranslationally modified to its biologically active form [139]. It is a member of bacteriocins, classified as a Type A (I) lantibiotic [140], identified in 1928 in fermented milk cultures [6]. It contains the hydrophobic residues at the N—terminus and hydrophilic residues at the C—terminus (Figure 9a) [138], five thioether rings and four amino acids, usually not found in nature: lanthionine (Lan), β-methyl lanthionine (MeLan), and two dehydrated amino acids—dehydroalanine (Dha) and dehydrobutyrine (Dhb) (Figure 9b,c) [141,142]. These amino acids result from posttranslational modification of serine, threonine, and cysteine [143]. Moreover, the thioether rings give nisin unique properties, including nanomolar antimicrobial activity, resistance against proteolytic degradation and high heat stability [135]. The first two thioethers rings can bind lipid II, the flexible hinge region together with the last two thioethers rings can flip into the membrane and create a pore [3]. Unmodified prenisin contains 57 amino acids: the first 23 from the leader peptide and the last 34 residues from the core peptide [144]. The leader peptide renders the propeptide inactive and must be cleaved for a nisin to gain antimicrobial activity [142]. Therefore, active nisin consists of only 34 amino acids [3].

Nisin is mainly produced by Gram-positive bacteria that include *Lactococcus* and *Streptococcus* species [7] (e.g., *Lactococcus lactis* (*L. lactis*) [137], *Streptococcus hyointestinalis* (*S. hyointestinalis*) [147],…). Various production strains also lead to different naturally occurring variants of nisin (nisin A, nisin Z, nisin F, …). The molecular weight of nisin depends on the production strain; usually, it is between 3.0 and 3.5 kDa [147]. This polypeptide has an amphipathic property [140], is cationic at neutral pH and has an isoelectric point above 8.5 [148]. Nisin has no absorbance at 280 nm due to the absence of aromatic amino acids [149].

Nisin has been approved by the Joint Food and Agriculture Organization World Health Organization (FAO/WHO, 1969), the US Food and Drug Administration (FDA, 1988) [7], the European Food Safety Authority (acceptable uptake of 0.13 mg/kg/day/person [150]) and the Food Standards Australia New Zealand [151]. It was generally regarded as safe (GRAS) [151,152]. So far, it is the only bacteriocin in the market allowed to be used as a food additive [153].

### 3.2. Isolation

Since the first discovery of nisin (nisin A) in fermented milk cultures, several natural and bioengineered variants of nisin have been identified [7,147], which differ in their structure and properties (solubility, chemical reactivity, and spectra) [154]. Up to now, there are eleven reported natural occurring nisin analogues: nisin A, nisin Z, nisin F, nisin Q, nisin H, nisin O A1-A3, nisin O A4, nisin U, nisin U2, nisin P, nisin J (Table 3), isolated from various bacterial genera such as *Lactococcus*, *Streptococcus*, *Staphylococcus*, and *Blautia*, located in dairy products, human gastrointestinal tract, bovine mammary secretions, human skin microflora, porcine intestine, an alimentary tract of ruminants, fish gut and river water in Japan [7,155]. Nisin analogues from the same genera are more like each other than analogues from different genera. Nisin A and nisin Z are both isolated from *L. lactis*, found in dairy products, and differ only in one amino acid at position 27; histidine (His) in nisin A is substituted with asparagine (Asp) in nisin Z (Table 3, highlighted in yellow). This substitution mainly affects the solubility of the polypeptide. It causes nisin Z to be more soluble at neutral pH than nisin A due to a more polar side chain of the Asp in comparison to His at neutral pH; it has minimal effect on antimicrobial activity, resistance to pH changes, sensitivity to proteolytic enzymes and thermal stability [149,155]. Nisin F differs from nisin A due to Asp and valine (Val) at positions 27 and 30. Nisin Q differs in comparison to nisin A in three amino acids at four positions: valine (Val, in position 15 and 30), leucine (Leu, in position 21), and asparagine (Asp, in position 27) [155]. Nisin O (A1-A3 and A4), nisin U and U2, and nisin P are shorter than previously described nisin analogues; they contain 33, 32, and 31 amino acids, respectively. With 35 amino acids, nisin J is the longest natural nisin analogue identified to date [147].

Aside from natural nisin analogues, the bioengineered forms of nisin have been developed in the last twenty years by genetic modification tools [156], with an attempt to alter the solubility, stability and efficiency of nisin. A large number of generated bioengineered forms of nisin revealed that modifying amino acids at the hinge-region (three amino acids asparagine-methionine-lysine at position 20–21–22 in the center of the peptide, Figure 9a) and at position 29, respectively, displayed an essential role in enhancing activity against Gram-negative bacteria, and both Gram-positive and Gram-negative pathogens [7,156]. Nisin A K22T, A N20P, A M21V, A K22S, A S29A, A S29D, A S29E, A S29G, Z N20K and Z M21K are some genetically modified nisin derivatives with changes in those positions and more significant activity against foodborne and clinical pathogens [6,7,156]. The names indicate the substitution position and the replaced amino acid; for example, nisin A K22T means that the amino acid sequence is the same as in Nisin A, the only difference is at position 22, where lysine (K) is substituted with threonine (T). Nisin derivative Z N20K and Z M21K showed enhanced activity against Gram-negative bacteria, including *Shigella*, *Pseudomonas* and *Salmonella* species, and displayed more significant thermal stability and solubility at neutral or alkaline pH [7]. Nisin A K22T exhibit enhanced activity against human and bovine pathogen *Streptococcus agalactiae* (*S. agalactiae*). Nisin A M21V showed enhanced antimicrobial activity against medically significant pathogens, including heterogenous Vancomycin intermediate *S. aureus* (hVISA), methicillin-resistant *S. aureus* (MRSA), *Clostridium difficile* (*C. difficile*), *S. agalactiae* and *Listeria monocytogenes* (*L. monocytogenes*). The S29G and S29A nisin variants showed enhanced activity against Gram-positive and Gram-negative pathogens, differentiating them from all nisin derivatives generated to date [156].

### 3.3. Bioactivity

**Figure 10:**
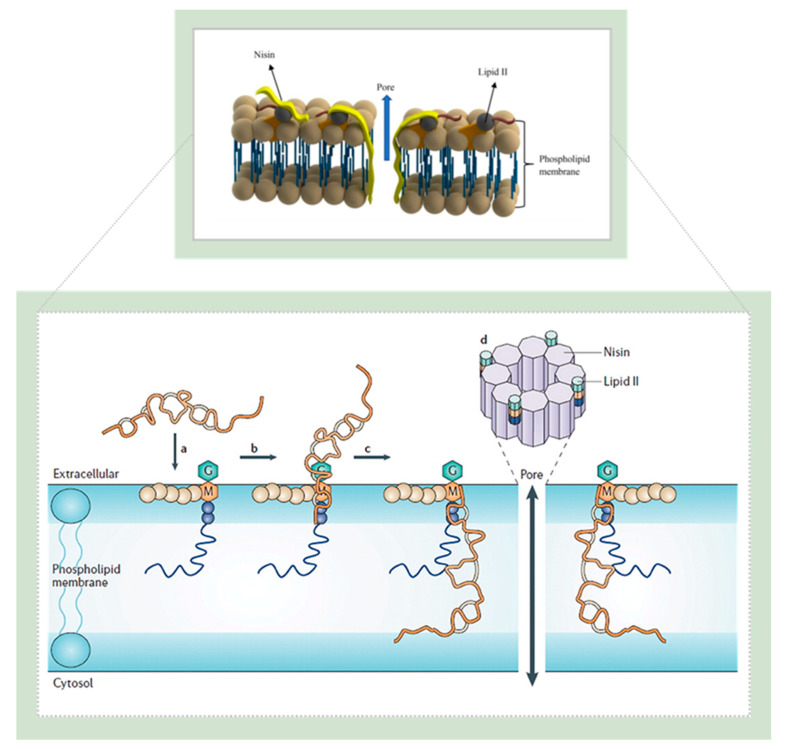
Schematic representation of the bactericidal mechanism of nisin: (**a**) nisin reaches the bacterial membrane; (**b**) adsorption of nisin to docking molecule (lipid II) via electrostatic interactions; (**c**) stable transmembrane orientation of nisin (cationic region of nisin interact with the negatively charged phospholipid heads, while the hydrophobic region of nisin interacts with the membrane core); (**d**) assembly of nisin-lipid II pore complex (consisting of 4 lipids II and 8 nisin molecules) (Reproduced with permission from [71,149], Elsevier, Amsterdam, The Netherlands, 2019 and Taylor & Francis, Abingdon, UK, 2016).

Nisin is known for its broad-spectrum of antibacterial activity against a wide range of Gram-positive bacteria [7,140], even better than conventional antibiotics [157], due to its stability at a higher temperature, tolerance to low pH, and dual-mode of antimicrobial activity [6]. The latter includes binding of nisin molecule to an essential precursor for bacterial wall biosynthesis (the lipid II) through electrostatic interaction between the positively charged nisin and the negatively charged membrane phospholipids. This results in the formation of the complex within the bacterial cell membrane, which creates 2 nm wide pores, thus preventing the growth of the peptidoglycan network and increased membrane permeability, leading to leakage of essential cellular components, and eventually to cell death (Figure 10) [71,139,149,158,159].

Nisin is active against a wide variety of Gram-positive *Lactococcus*, *Enterococcus*, *Streptococcus*, *Staphylococcus*, *Listeria* and *Micrococcus* bacterial strains, as well as the vegetative forms and outgrowing spores of *Bacillus* and *Clostridium* species [138,142,158]. The Gram-negative bacteria (e.g., *E. coli*) are usually resistant to nisin due to their outer lipopolysaccharide membranes, which act as a barrier/shield and impede its access to the cytoplasmic membrane [160,161]. Additionally, nisin shows no inhibitory activity against yeast cells, filamentous fungi and viruses [149]. However, many studies [6,7,142,156,158,160,162,163,164] demonstrate that bioengineered variants of nisin, high purity nisin, nisin-antibiotics, nisin-chelating agents (e.g., EDTA), nisin-inorganic nanoparticles (silver, gold, magnesium oxide,…) or other outer membrane destabilizing component/processes (e.g., heat treatment, freezing) could also be effective against Gram-negative bacteria.

Required nisin concentration for efficient bacteria inhibition depends on several parameters, such as pH, heat treatment intensity, storage time and storage conditions. Aqueous solubility and structural stability of nisin are also pH dependent. The antimicrobial activity, solubility, and thermal stability of nisin are higher at acidic pH and deactivate under alkaline conditions due to irreversible structural changes of the nisin molecule. Nisin has higher antimicrobial activity in a liquid medium than a solid medium. Nisin is highly stable at low temperatures (e.g., during freezing), but undergoes a loss of activity during long-time heating. Proteolytic enzymes such as pancreatin, α-chymotrypsin and ficin can inactivate nisin due to their ability to break down the peptide chain of nisin. Other enzymes such as trypsin, pepsin and carboxypeptidase have no significant effect on its antimicrobial effect. The antimicrobial activity of nisin is also inhibited by the titanium dioxide and sodium metabisulphite due to the oxidation of disulfide bridges in the nisin molecule [149,165].

### 3.4. Immobilization Strategies

**Figure 11:**
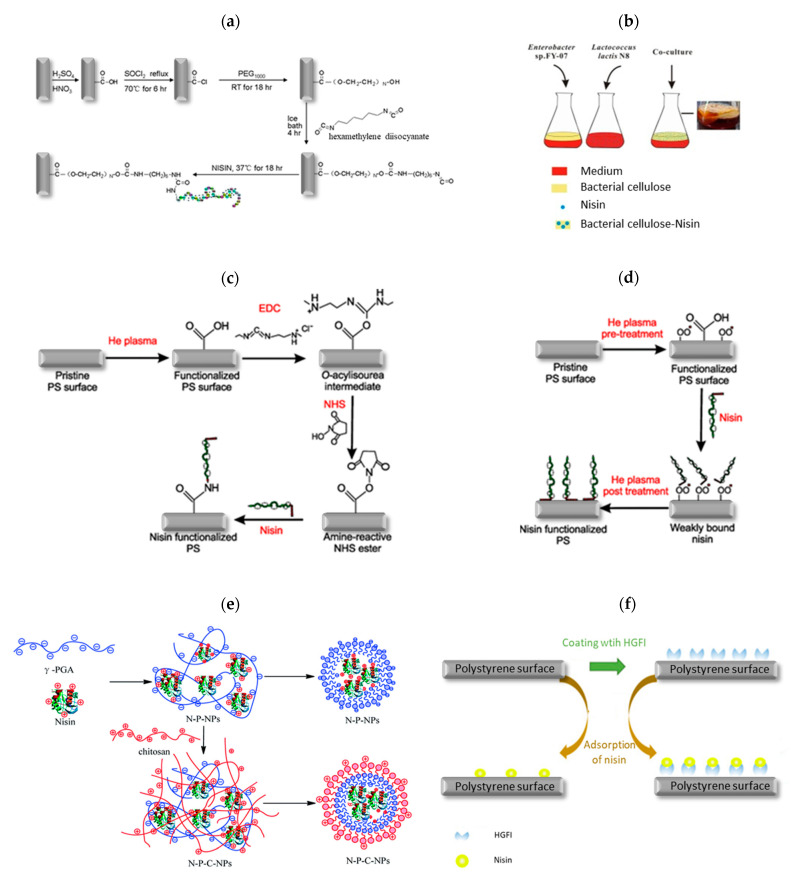
Schematic illustration of immobilization methods of nisin: (**a**) covalent immobilization onto multi-walled carbon nanotubes with PEG_1000_ as a linker and hexamethylene diisocyanate as a crosslinking agent (Adopted with permission from [176], RSC, 2011); (**b**) co-culture fermentation of nisin-producing (*Lactococcus lactis* N8) and bacterial cellulose-producing (*Enterobacter* sp. FY-07) bacteria (Adopted with permission from [153], Elsevier, Amsterdam, The Netherlands, 2021); (**c**) covalent immobilization onto plasma-treated, EDC/NHS ester functionalized polystyrene sheets (Adopted with permission from [138], RSC, 2017); (**d**) covalent immobilization onto plasma-treated polystyrene sheets (Adopted with permission from [138], RSC, 2017); (**e**) nisin loaded chitosan-poly-γ-glutamic acid nanoparticles (encapsulation) (Reproduced with permission from [179], RSC, Cambridge, UK, 2016); (**f**) adsorption of nisin on blank and HGFI-coated polystyrene surface together with antimicrobial activity of both surfaces (Adopted with permission from [9], Elsevier, Amsterdam, The Netherlands, 2021).

**Figure 12:**
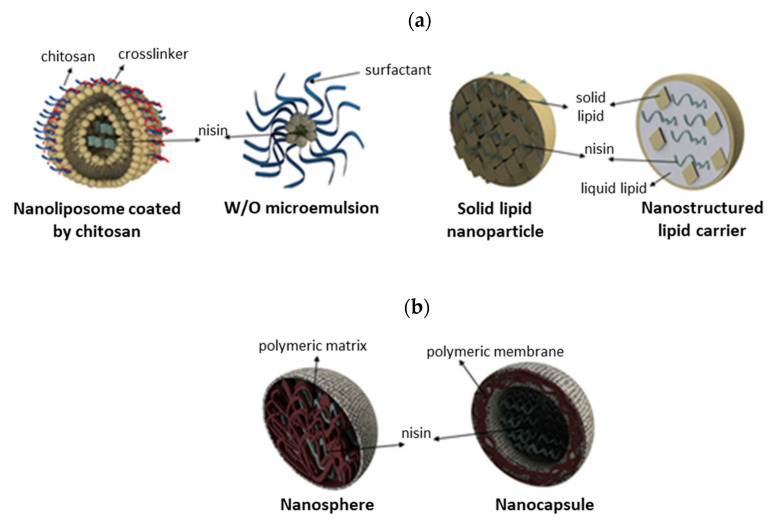
Scheme of (**a**) lipid-based nanoparticles and (**b**) biopolymeric nanoparticles for encapsulation of nisin (Adopted with permission from [71], Elsevier, Amsterdam, The Netherlands, 2019).

Various immobilization methods have been developed to protect nisin from environmental stresses, degradation by biological fluids or biocomponents (i.e., proteolytic enzymes) or deactivation under alkaline conditions [4,165], including covalent immobilization, encapsulation, entrapment, adsorption and co-culture fermentation, summarized in Table 4 and Figure 11. Most of the reported strategies for nisin immobilization required special pre-treatment of used support material/carrier, chemical modifications, crosslinking agents (carbodiimide/N-hydroxysuccinimide (EDC/NHS), hexamethylene diisocyanate, glutaraldehyde,…) or a variety of other spacer molecules to obtain a composite with optimal, target-directed antimicrobial action against pathogenic bacteria [4,9,161]. In recent years, great emphasis has been placed on developing innovative nano-engineered approaches and nanostructured materials with enhanced antimicrobial activity in comparison to free nisin, including lipid-based nanoencapsulated nanoparticles (nanoliposomes, nanoemulsions, nanomicelles, solid lipid nanoparticles and nanostructured lipid carriers, Figure 12a), polymeric-based nanoencapsulated nanoparticles (nanocapsule and nanosphere, Figure 12b) and nanofibers [71,166]. Natural and synthetic materials studied as carrier or support material for immobilization of nisin includes liposomes [164,167], silica xerogels [168], polystyrene sheets [138], polyethylene oxide brush layer [169], soy lecithin liposomes [170], bacterial cellulose nanocrystals [151], chitosan nanoparticles [171], alginate beads [172] or a mixture of pectin-chitosan microcapsules [165], alginate-starch microcapsules [173], alginate-pectin microbeads [174] or chitosan-alginate microparticles [175],... having antimicrobial activity against various Gram-positive and Gram-negative bacteria (Table 4).

**Table 4:** Immobilization methods of nisin and antimicrobial activity against Gram-positive and Gram-negative bacteria.

Immobilization Method	Carrier/Support Material	Crosslinking Agent or Initiator	Antimicrobial Property against	References
Covalent immobilization	Multi-walled carbon nanotubes grafted with poly(ethylene glycol) (PEG_1000_)	Hexamethylene diisocyanate	*E. coli,* *P. aeruginosa,* *S. aureus,* *B. subtilis*	[176]
	Polystyrene (PS) sheets	Atmospheric-pressure plasma	Gram-positive *S. aureus* and *L. monocytogenes*	[138]
	Poly(vinyl alcohol) films	Glutaric acid	Gram-positive *S. aureus* and Gram-negative *E. coli*	[177]
	Sodium alginate/gelatin wet-spun porous fibers	GA	*S. aureus*	[4]
	N-succinyl chitosan films	EDC	*S. aureus*, *E. coli*, *S. enteritidis*, *Pseudomonas tolaasii* (*P. tolaasii*)	[178]
Encapsulation	Silica xerogel	/	*B. cereus*, *L. monocytogenes*, *S. aureus*, *E. coli*, *S. enterica*	[168]
	Bacterial cellulose nanocrystals	/	*L. rhamnosus* LBM1	[151]
	Chitosan nanoparticles	/	*E. coli* and *S. aureus*	[171]
	Ca-alginate microparticles	/	*Brochothrix thermosphacta* (*B. thermosphacta*) 7R1	[172]
	Pectin-chitosan microcapsules	/	*S. aureus*, weak bactericidal effect on *E. coli* under acidic conditions	[165]
	Alginate-starch microcapsules	/	*Pediococcus acidilactici* (*P. acidilactici*) UL5	[173]
	Chitosan-alginate microparticles	/	N/A	[175]
	Chitosan-poly-γ-glutamic acid nanoparticles	/	*E. coli* and *L. monocytogenes*	[179]
	Phosphatidylcholine liposomes containing chitosan or chondroitin sulfate	/	*L. monocytogenes*	[180]
	Soybean lecithin or Phospholipon^®^ liposomes	/	*L. monocytogenes*, *Clostridium perfringens* (*C. perfringens*) *and Bacillus cereus* (*B. cereus*)	[181]
Co-encapsulation	Phosphatidylcholine (PC) nanoliposomes coated with pectin or polygalacturonic acid	/	*L. monocytogens, Salmonella* Enteritidis (*S.* Enteritidis)	[167]
	Phosphatidylcholine (PC) nanoliposomes	/	*L. monocytogenes*, *S.* Enteritidis, *E. coli and S. aureus*	[164]
Adsorption	Low density polyethylene films treated with acrylic acid	/	*Listeria innocua* (*L. innocua*)	[182]
	Montmorillonite suspension	/	*E. faecium* C1	[183]
	Polyethylene oxide brush layers	/	N/A	[169]
	HGFI (class I hydrophobin)-coated polystyrene films	/	*S. aureus*	[9]
	ZnAl layered double hydroxides nanohybrids	/	N/A	[184]
Co-culture fermentation	None		*S. aureus*, *E. coli*	[153]
Entrapment	Polyethylene oxide brush layer	/	*Pediococcus pentosaceous* (*P. pentosaceous*)	[185]
	PET (polyethylene terephthalate) woven fabrics with thin alginate coating	/	*S. aureus*	[186]
	Poly-ethylene-co-vinyl acetate films	/	*Staphylococcus epidermidis* (*S. epidermidis*) ATCC 35984, *S. aureus* 815 and *L. monocytogenes* ATCC 7644	[187]
	Guar gum gel (biogel)	/	Canine oral enterococci collection (including *E. faecalis* and *E.* *faecium*)	[188]

**Figure 13:**
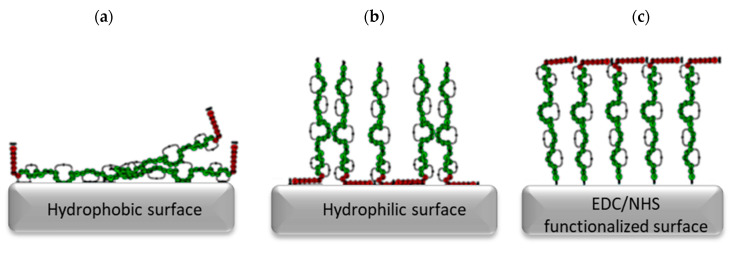
Proposed orientation of nisin on (**a**) hydrophobic surface, (**b**) hydrophilic surface and (**c**) with EDC/NHS functionalized surface (Adopted with permission from [138], RSC, 2017).

However, different hydrophilic/hydrophobic surface properties of these carriers affect the orientation of the nisin (Figure 13). It is proposed that the hydrophobic region of the nisin binds to the hydrophobic surface, leading to the reduced number of hydrophobic regions available to interact with the bacterial cell membrane. Similarly, the hydrophilic region of nisin binds to the hydrophilic surface, allowing the hydrophobic region to interact with the bacterial cell membrane [138]. Furthermore, nisin reacts with EDC/NHS functionalized surface through its amine group at the N-terminus, which could cause inefficient adsorption to the carrier due to steric barriers of the hydrophobic region [138].

### 3.5. Applications

Nisin’s properties, such as inhibitory efficiency against a wide range of microorganisms, low probability of developing microbial resistance, no effect on the normal microbiota of the intestine, non-toxicity, colourless and tasteless, enable its use in both the biomedicine and food industry [137,189], especially in the second segment, where use as food bio preservative is already much exhausted [190]. Nisin is used to preserve pasteurized milk, aged cheeses, canned soups, juice, meat and vegetables [71,149]. It shows a better choice for prolonging the shelf life of meat (Tan sheep meat) in comparison to preservative potassium sorbate due to reduced nutrient loss [191]. Furthermore, it can be combined with other pasteurization preservation treatments to increase inhibition effectiveness against heat-resistant spore-former and extend the food shelf life [149]. As a food additive, it is assigned as E234 [149] and has been approved for use in over 60 countries around the world as a natural agent to prevent food spoilage due to its low toxicity or non-toxicity, high efficiency [153], thermal stability, and colourless and tasteless properties [157]. Saini et al. [150] studied covalent immobilization of nisin on the surface of TEMPO-oxidized CNF and thus developed antimicrobial films, which could be used as active food packaging. Nisin was also studied to develop impedimetric label-free biosensors for bacterial contamination detection of *Salmonella* spp. [192].

**Figure 14:**
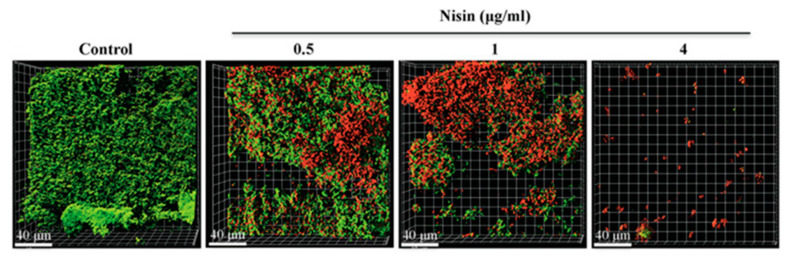
Confocal microscopy images of the influence of nisin concentration on oral biofilm formation under the controlled microfluidic model system. A green signal indicates viable live cells (Syto 9) and a red signal indicates damaged/dead cells (propidium iodide). No biofilm was observed at a nisin concentration of 4 μg/mL (Reproduced with permission from [140], Meta UCL, 2015).

In light of biomedical potential, the nisin already demonstrates promising results as an alternative to traditional antimicrobial therapeutics due to its activity against specific (antibiotic-resistant bacterial) pathogens and disease conditions, particularly concerning mastitis in lactating women and dairy cows (inhibition of *S. aureus* and *S. epidermidis* [193,194,195,196,197,198]), respiratory infections (inhibition of *S. aureus* [199]) and skin infections, e.g., atopic dermatitis [200] and MRSA skin infections (inhibition of *S. aureus*) [147,201,202,203,204]. It can be used either as a single agent or in combination with other agents [7,157,189,201,205]. Furthermore, it showed potential in oral diseases, such as caries and periodontal diseases, due to inhibition of oral bacteria, including *Streptococcus sanguinis* (*S. sanguinis*), *Streptococcus sobrinus* (*S. sobrinus*), *Streptococcus gordonii* (*S. gordonii*), *P. gingivalis*, *Prevotella intermedia* (*P. intermedia*), *A. actinomycetemcomitans* and *Treponema denticola* (*T. denticola*) [140,206,207]. Shin and co-workers [140] studied nisin’s antimicrobial efficiency against the formation of saliva-derived multi-species oral biofilms. They reported on reduced biofilm biomass in a dose-dependent manner (Figure 14); no apoptotic changes of human oral cells were observed at nisin concentration <200 μg/mL [140]. Nisin also has the potential to control periodontal disease in dogs [208].

Additionally, nisin has been studied as a possible anticancer agent due to the multidrug resistance of cancer cells and drastic side effects of traditional chemotherapeutics [209,210]. Hosseini and co-workers reported a significant decrease in the growth rate of SW480 colorectal cancer cell line after being treated with nisin [211]. Similar conclusions are reported by Tavakoli et al. [212]. Nisin also showed a significant efficiency as an adjuvant to conventional chemotherapeutic agents. Preet et al. studied synergism between doxorubicin, a chemotherapeutic drug traditionally used to treat breast cancer, lymphoma, bladder cancer, acute lymphocytic leukemia [8], and nisin against skin carcinogenesis [209]. They reported on augmented anticancer activities when both these agents were used in conjunction with each other [209]. Rana and colleagues studied the possible use of a 5-fluorouracil-nisin combination as a topically applied chemotherapeutic drug against skin cancer [210]. They observed faster clearance of tumors and a reduced dose of 5-fluorouracil when a 5-fluorouracil-nisin combination was used [210]. Joo et al. reported on increased cell apoptosis and decreased cell proliferation at head and neck squamous cell carcinoma by nisin treatment [159]. Furthermore, nisin A has been demonstrated to have a potential for treating nonhealing wounds, as it increases the mobility of skin cells, dampens the effect of lipopolysaccharide and proinflammatory cytokines, and decreases bacterial load in the wound [157].

## 4. Combination of Bioactive Compounds

Simultaneous use of (bio)active agents is common practice to collect multiple activities and even augment their efficiency to a higher level than their simple sum. The use of enzymatic mixtures, comprising enzymes with wide diversity in the reactions they are catalyzing, is one frequent case of simultaneous use of multiple bioactive compounds. Moreover, the ˝crude enzymatic cocktails˝ (as crude bromelain itself) are more frequently present in nature than a single, specific type. Aside from simple mixtures, more than one enzyme’s co-immobilization was found very efficient in terms of product yield and thermal stability increment, as present in the triple enzyme system [213]. Another example is antibiotics, where the combined therapy utilizing more than one antibiotic at the time is practiced in particular cases in order to broaden the antibacterial spectrum, to treat the polymicrobial infections, to obtain synergistic effect bringing higher efficiency at lower doses and finally, to tackle the emergence of bacterial resistance [214].

The bacteriocin nisin offers a range of advantageous features that include protease and heat stability; its efficacy can be further boosted via combination with other antimicrobials or membrane-active substances. Nisin demonstrates synergistic activity with the antibiotics colistin and clarithromycin against *P. aeruginosa* [215] with ramoplanin and other-β-lactam antibiotics against many strains of MRSA and VRE [216] with penicillin, streptomycin, chloramphenicol and rifampicin against *Pseudomonas fluorescens* [217]. Combinations of derivatives nisin V + penicillin or nisin I4V + chloramphenicol had an enhanced inhibitory effect against *S. aureus* SA113 and *S. pseudintermedius* DSM21284, respectively, compared to the equivalent nisin A + antibiotic combinations or when each antimicrobial was administered alone [218].

Reported studies demonstrate that such mixtures boost the antimicrobial action, but the same does not introduce new bioactive functions. One-pot (co-immobilisation, simultaneous immobilisation), or successive immobilization of bioactive compounds, together with diverse immobilisation strategies, all together present modalities to be used in obtaining a multi-active system including different types of bioactive compounds. Such an example is a two-step polydopamine-based surface modification strategy, used to co-immobilize an antimicrobial peptide Palm and an enzyme targeting an important component of biofilm matrix (DNase I). This immobilization approach imparted polydimethylsiloxane surfaces with both anti-adhesive and antimicrobial properties against the adhesion of relevant bacteria as single and dual-species, with excellent stability and biocompatible and anti-biofilm properties, holding, therefore, great potential in the development of catheters able to prevent the catheter-associated infections [219].

To date, the co-immobilization of bromelain and nisin as proteolytic enzymes and protease-resistant antimicrobial peptide, respectively, has not been trialled. Aside from obstacles anticipated to such an experimental design, the potential success may offer a merge of an extensive portfolio of bioactive functions brought by both components. Both components are complementary in many terms, including the type of bacteria they are acting against, i.e., Gram-positive for nisin and Gram-negative for bromelain.

## 5. Conclusions and Prospects

Bromelain and nisin are undoubtedly among more perspective, natural bioactive components with outstanding potential in biomedicine due to diverse therapeutic benefits, demonstrated by several research groups in the recent decade. In vitro studies of bromelain and nisin show their potential in human medicine and healthcare, in the treatment of skin infections, caries, periodontal diseases, and many other conditions. Importantly, the bromelain shows promise within several in vitro studies involving cancer cell lines, yet, the clinical trials in this segment are in a premature stage, with only two examples at the moment (one for treatment of solid tumors in advanced stage of lung, breast, colon, ovary, cervix, uterus, prostatic, and liver and second for treatment of Pseudomyxoma Peritonei, Peritoneal Cancer, Mucinous Adenocarcinoma and Mucinous Tumor) [220]. The plant extract bromelain interacts with several biological processes that lead to its multi-action bioactivity, including antimicrobial, anti-inflammatory, anticarcinogenic and antithrombotic activity. Unlike bromelain, which has already gained FDA approval in topical product NexoBrid, the nisin is only approved as a food additive despite its effectiveness against drug-resistant organisms also in biomedical research. Nonetheless, much effort has been devoted to widening the nisin efficiency from Gram-positive bacteria towards Gram-negative bacteria, where biotechnological approaches or combination with other components (antibiotics, inorganic nanoparticles, chelating agents, …) have been applied, which paves its way towards use in more demanding clinical set-ups. Further, the production of different variants (from native and gene-modified bacterial species) with a high degree of purity, securing the safeness of final products are evidencing recognized the potential of this bioactive compound.

To the best of our knowledge, the synergistic action of both bioactive components is yet to be explored as an attractive topic. Before going ahead with a cost-demanding clinical translation of bromelain- or nisin-containing materials developed in a lab, much remains to be learned, particularly about different variants and combinations with conventional antibiotics and cancer drugs, their complex mechanism of action on the human body and pathogens, consequences of long-term clinical trials and choosing suitable optimized immobilization method with high immobilization yield and secured activity/efficiency. As said, most data for bromelain and nisin demonstrated an in vitro efficiency, and the extrapolation of in vitro to in vivo outcome is not that straightforward, yet, same present a solid background, important in future translation in a clinic. With all this, it will be possible to offer novel, safe and efficient natural therapeutic solutions to our society without significant risks to developing resistance in pathogenic organisms and cells.

## Figures and Tables

**Table 3:** Primary structures of nisin natural analogues. The changes in amino acids compared to nisin A are highlighted in yellow (not valid for Nisin J).

**Natural nisin analogues represented with a primary structure** (Reproduced with permission from [147,155], ASM Journals, Washington, USA, 2020 and Springer Nature, London, UK, 2020)		**Production strain** [7,155]	**Molecular weight [Da]** [147]
Nisin A	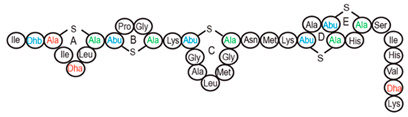	*Lactococcus lactis* (dairy products)	3354
Nisin Z	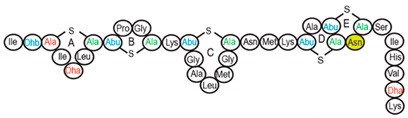	*Lactococcus lactis* NIZO 22,186 (dairy products)	3331
Nisin F	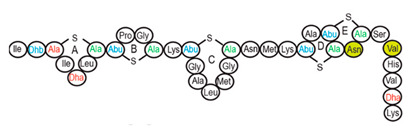	*Lactococcus lactis* F10 (fish gut)	3315
Nisin Q	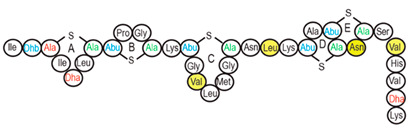	*Lactococcus lactis* 61–14 (Japanese river water)	3327
Nisin H	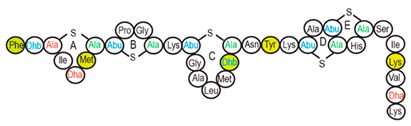	*Streptococcus hyointestinalis* (porcine intestine)	3453
Nisin O A1-A3	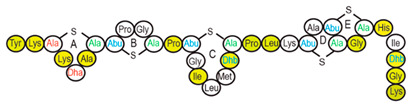	*Blautia obeum* A2–162 (human gastrointestinal tract)	3546
Nisin O A4	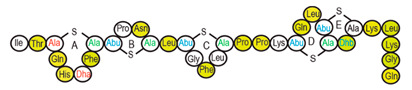	*Blautia obeum* A2–162 (human gastrointestinal tract)	3259
Nisin U	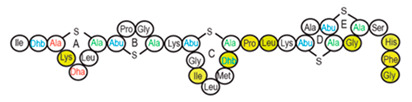	*Streptococcus uberis* 42 (bovine mammary secretions)	3029
Nisin U2	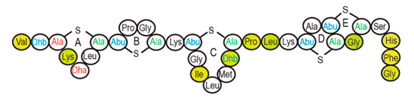	*Streptococcus uberis* D536 (bovine mammary secretions)	3015
Nisin P	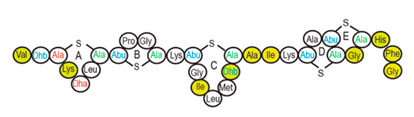	*Streptococcus gallolyticus subsp. Pasteurianus* (alimentary tract of ruminants)	2989
Nisin J	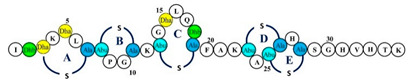	*Staphylococcus capitis* APC 2923 (human skin)	3458

## Abbreviations

AMP	antimicrobial peptide
APTES	(3-aminopropyl)-triethoxysilane
Asp	asparagine
BBT	bromelain-based treatment
CDTA	cyclohexane-1,2-diaminoetetraacetic acid
COX-2	prostaglandin-endoperoxide synthase 2
CP	cysteine proteinase
DETA	diethylenetriamine
Dha	dehydroalanine
Dhb	dehydrobutyrine
ECM	extracellular matrix
EDC	1-ethyl-(3-dimethylaminopropyl) carbodiimide
EDTA	ethylenediaminetetraacetic acid
EMA	European Medicines Agency
GA	glutaraldehyde
HEDTA	hydroxyethyl ethylenediamine triacetic acid
IL	interleukin
Lan	lanthionine
LD_50_	lethal dose
Leu	leucine
K_m_	Michaelis–Menten constant
MBC	minimum bactericidal concentration
MeLan	β-methyl lanthionine
MIC	minimum inhibitory concentration
MRSA	methicillin-resistant *Staphylococcus aureus*
MSN	mesoporous silica nanoparticles
NCBI	National Center for Biotechnology Information
NF-κB	Nuclear factor kappa B
NHS	N-hydroxysuccinimide
PC	phosphatidylcholine
PEG	poly(ethylene glycol)
PEHA	pentaethylenehexamine
PET	polyethylene terephthalate
PGE-2	prostaglandin E_2_
PGI-2	prostaglandin I_2_
pI	isoelectric point
PS	polystyrene
SEM	scanning electron microscopy
SPRA	self-assembly PEGylation retaining activity
TEPA	tetraethylenepentamine
TETA	triethylenetetramine
TNF	tumour necrosis factor
TXA-2	thromboxane A2
V_max_	maximum reaction velocity
Val	valine
